# Upregulation of HOTTIP and Its Potential Role in Monitoring Exercise Adaptation

**DOI:** 10.3390/ijms26168086

**Published:** 2025-08-21

**Authors:** Agnieszka Mołoń, Dominika Podgórska, Artur Płonka, Wojciech Bajorek, Wojciech Czarny, Paweł Król, Rafał Podgórski, Marek Cieśla

**Affiliations:** 1 Laboratory of Diagnostic and Clinical Epigenetics, Faculty of Medicine, University of Rzeszów, 2A Kopisto Ave., 35-959 Rzeszów, Poland; agmolon@ur.edu.pl; 2 Department of Rheumatology, Faculty of Medicine, University of Rzeszów, 16C Tadeusza Rejtana Ave., 35-959 Rzeszów, Poland; 3 Faculty of Physical Culture Sciences, University of Rzeszów, 16C Tadeusza Rejtana Ave., 35-959 Rzeszów, Poland; 4 Department of Medicinal Chemistry and Metabolomics, Faculty of Medicine, University of Rzeszów, 16C Tadeusza Rejtana Ave., 35-959 Rzeszów, Poland; rpodgorski@ur.edu.pl

**Keywords:** long non-coding RNA, molecular biomarker, professional athletes

## Abstract

Athletic performance is modulated by a complex interaction of physiological, environmental, and genetic factors, with regular exercise triggering molecular changes that influence gene expression and tissue adaptation. Despite growing knowledge, the underlying molecular mechanisms remain only partially understood, highlighting the need for precise biomarkers to monitor training-induced physiological adaptations. Long non-coding RNAs (lncRNAs) regulate cellular processes, including adaptation to physical exercise. Twelve healthy elite female volleyball players (mean age 27 ± 5.4 years) participated in the study. This study evaluated the expression of selected lncRNAs (SNHG4, SNHG5, PACERR, NEAT1, HIX003209, and HOTTIP) during a 10-week training program and evaluated their potential as biomarkers of training adaptation. Blood samples were collected before and after the training period. LncRNA expression was measured by quantitative polymerase chain reaction. HOTTIP exhibited an increase in expression after training (over sixfold change, *p* = 0.009, adjusted *p* = 0.024) and demonstrated high diagnostic accuracy (AUC = 0.917), which improved to 0.97 when combined with creatine kinase. Other lncRNAs showed no significant changes, although a correlation between HOTTIP and SNHG4 was noted. HOTTIP is markedly upregulated following chronic exercise and, especially when combined with creatine kinase, shows promise as a molecular biomarker for monitoring training adaptation in elite female volleyball players.

## 1. Introduction

Athletic performance is influenced by the interplay of multiple factors, ranging from physiological condition, training status, and nutritional state to environmental, psychological, and molecular determinants. Regular exercise induces a cascade of signaling events at both the extracellular and intracellular levels. These changes significantly affect the expression of genes involved in regulatory processes, mitochondrial biogenesis, angiogenesis, and metabolism of cardiac and skeletal muscle, as well as tissue regeneration, remodeling, and inflammatory responses [1].

Basic research enables the identification of biochemical markers associated with inflammation and adaptive responses induced by physical exercise and training status. Among the most prominent of these are creatine kinase (CK), lactate dehydrogenase, cortisol (CORT), and high-sensitivity C-reactive protein (hs-CRP) [2,3,4,5]. Although the complexity of these processes is increasingly understood, their molecular mechanisms remain incompletely elucidated. Addressing this challenge may require the development and identification of biomarkers that provide detailed characterization of physiological adaptations and precise assessment of exercise-induced changes. The foundation of the adaptive response to physical exertion—aimed at enhancing metabolic efficiency, muscle contractile activity, and oxidative capacity—is alterations in mRNA expression profiles. These changes are accompanied by variations in the levels of proteins encoded by numerous genes that regulate mitochondrial function and cellular energy metabolism [6]. However, this expression does not occur in isolation but is influenced by other regulatory mechanisms related to epigenetics [7,8]. Epigenetic processes regulate the level of gene expression without changing the genetic information contained in the genetic code. Their main mechanisms include DNA methylation, histone protein modifications, and the influence of non-coding RNA. Over the years, the concept has been well established that gene expression is governed by a complex transcriptional network in which various non-coding RNAs (ncRNAs) play a crucial regulatory role [9,10]. Findings from the Human Genome Project revealed that protein-coding genes constitute only ~2% of the human genome, shifting scientific focus toward the regulatory roles of non-coding sequences [11,12].

NcRNAs are broadly classified into housekeeping RNAs (e.g., ribosomal RNAs and transfer RNAs), small ncRNAs (including transcripts less than 300 nt, e.g., microRNAs (miRNAs), small nucleolar RNAs, and PIWI-interacting RNAs), and long ncRNAs (lncRNAs; including more than 300 nt), including long intergenic ncRNAs, natural antisense transcripts (NATs), and circular RNAs (circRNAs) [13]. These molecules are critical in gene expression regulation and other fundamental cellular processes.

LncRNAs, first described in 1991 [14], are now recognized as important regulators of gene expression. Over 58,000 distinct lncRNAs have been identified in the human transcriptome, although many remain uncharacterized [13,15]. These molecules function as scaffolds, guides, or decoys for proteins and nucleic acids, and their expression is highly cell- and tissue-specific, as well as responsive to environmental stimuli, including physical activity. Emerging evidence suggests that exercise modulates lncRNA expression in an activity-specific manner. Some lncRNAs, such as NEAT1 and MALAT1, promote myoblast proliferation, whereas others, including H19 and linc-MD1, enhance differentiation [16,17]. Further research is needed to clarify how variables such as training type, sex, age, fitness level, and body composition affect lncRNA expression and contribute to exercise-induced physiological adaptations. HOTTIP interacts with H3K4me3 histones, facilitating mesenchymal tissue remodeling, angiogenesis, and transcriptional reprogramming. It also functions as a competing endogenous RNA, sequestering miRNAs that influence cell proliferation, migration, and apoptosis [18]. SNHG4 and SNHG5 are involved in muscle regeneration and metabolic homeostasis [19,20,21,22,23]. NEAT1 and HIX003209 are implicated in cellular stress response and hypoxia regulation, while PACERR controls COX-2 expression and inflammatory processes [17,24,25,26,27,28,29].

The aim of this study is to evaluate the association between the expression levels of six selected lncRNAs—SNHG4, SNHG5, PACERR, NEAT1, HIX003209, and HOTTIP—in response to prolonged intensive training loads and their relationship with training parameters among professional athletes. Additionally, the study aims to assess the potential of these lncRNAs as biomarkers of training effectiveness.

## 2. Results

### 2.1. Relationship Between Studied lncRNAs and Training Parameters

**Figure 1:**
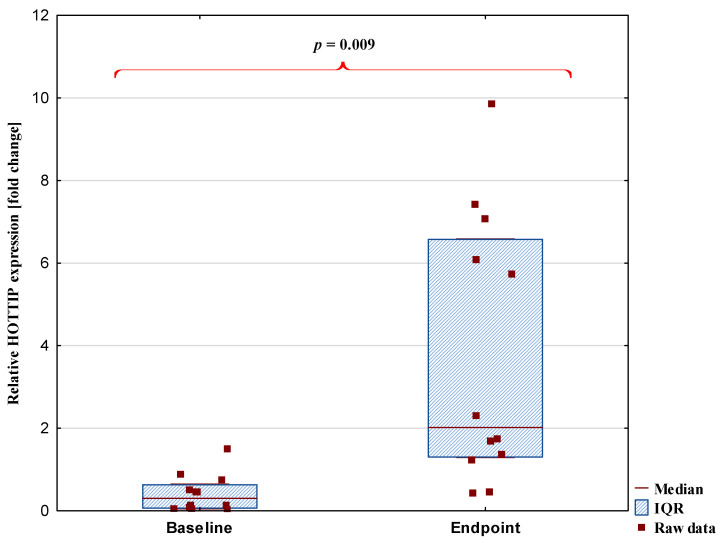
Difference in HOTTIP lncRNA expression levels between baseline and endpoint. Data are presented as median [interquartile range, IQR]. The *p*-value after correction for multiple testing was *p* = 0.024.

The expression of the studied lncRNAs was measured at a 10-week interval and at two time points: at the beginning (baseline) and end of this period (endpoint). A significant change in expression levels was observed only for the HOTTIP transcript. The median expression level of HOTTIP increased markedly from 0.30 [0.62–0.63] to 2.02 [1.30–6.57], representing more than a sixfold increase. Details were presented in Figure 1. The other analyzed lncRNAs, including SNHG4, SNHG5, NEAT1, HIX003209, and PACERR, did not show significant changes between the baseline and endpoint measurements. Changes in the expression levels of selected lncRNAs and biochemical parameters between baseline and endpoint are presented in Table 1. Creatine kinase levels increased by approximately 106.7% from baseline to endpoint (*p* = 0.001; adjusted *p* = 0.004), while cortisol concentrations decreased by approximately 35.8% (*p* ≤0.00; adjusted *p* = 0.002). The detailed absolute values, including means and standard deviations, have been reported in our previous study [5,30].

In most cases, the lncRNAs analyzed in this study did not exhibit significant correlations with training-related parameters. Detailed results are presented in Table 2 and Table 3. Notable baseline correlations were observed between SNHG4 and body fat percentage (%FAT) (r = 0.69), as well as between HIX003209 and cortisol levels (r = 0.62). At the endpoint, the lncRNA HIX003209 exhibited a strong negative correlation between changes in its expression levels and the percentage change in creatine kinase levels (r = −0.8).

Correlation analysis of relative lncRNA expression levels at baseline (Table 4) revealed positive associations between SNHG4 and HOTTIP (r = 0.89), as well as between SNHG4 and HIX003209 (r = 0.71). Additionally, a positive correlation was observed between HIX003209 and PACERR (r = 0.59). Following the training period (Table 5), the correlation structure among the analyzed transcripts demonstrated notable changes. The correlation between SNHG4 and HOTTIP remained (r = 0.70). Other correlations between targets were observed: SNHG4 and NEAT1 (r = 0.83), HOTTIP and NEAT1 (r = 0.58), and HOTTIP and PACERR (r = 0.59).

### 2.2. Assessment of Training Effectiveness Analysis Using ROC Curves

**Figure 2:**
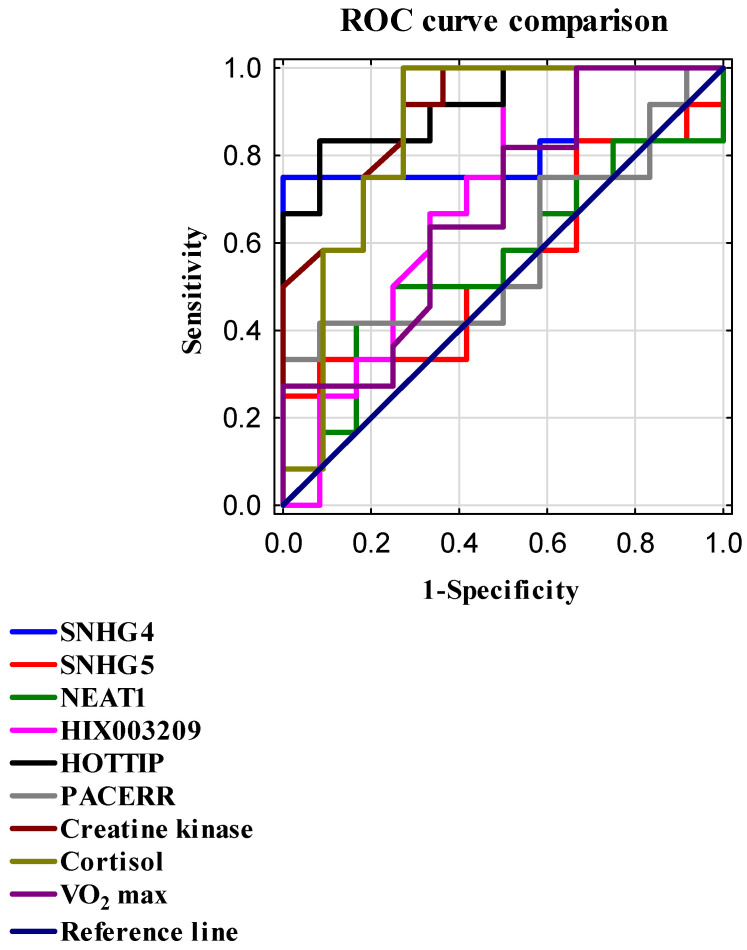
ROC curves were generated to compare the diagnostic performance of selected biomarkers and lncRNAs in assessing training effectiveness. The reference line (AUC = 0.5) indicates no discriminative ability. Sensitivity (true positive rate) is plotted on the *Y*-axis, while 1—specificity (false positive rate) is plotted on the *X*-axis. Both metrics are expressed as normalized values ranging from 0 to 1.

To evaluate the diagnostic capability of individual parameters, receiver operating characteristic (ROC) curve analysis was performed. For each marker, the area under the curve (AUC) and Youden’s index, as well as sensitivity and specificity at the optimal cutoff point, were determined; details are presented in Table 6 and Figure 2. Among the studied lncRNAs, the markers HOTTIP and SNHG4 demonstrated significant diagnostic value. HOTTIP exhibited the highest AUC of 0.917, with sensitivity of 83%, specificity of 92%, and a Youden’s index of 0.75. Similarly, SNHG4 showed an AUC of 0.785, sensitivity of 75%, specificity of 100%, and an identical Youden’s index of 0.75. High AUC values were also observed for CK (0.894; sensitivity 92%; specificity 73%; Youden’s index 0.64) and cortisol (0.856; sensitivity 100%; specificity 73%; Youden’s index 0.73).

**Figure 3:**
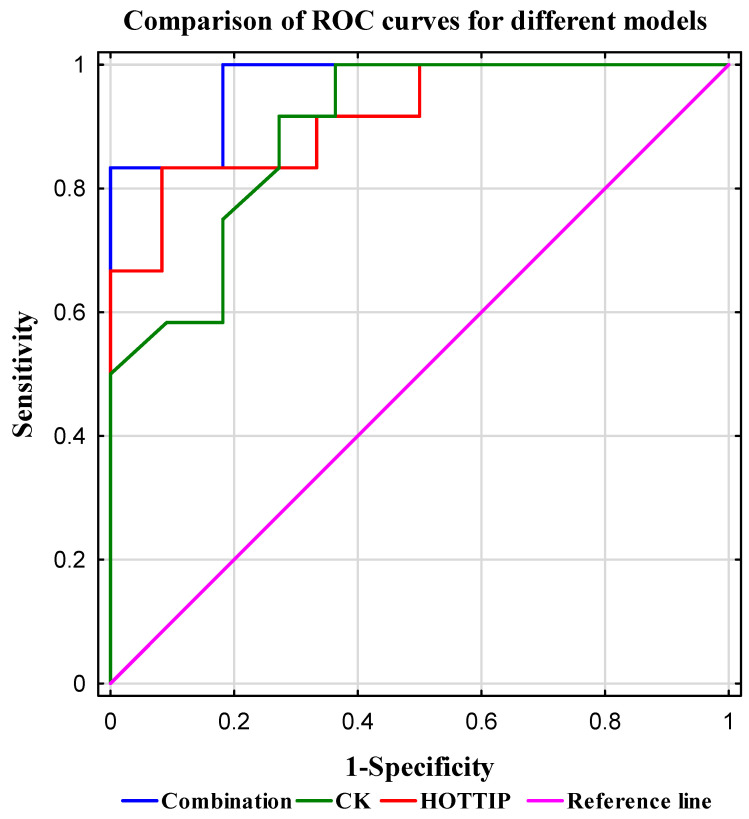
Comparison of ROC curves for different models. The plot shows the sensitivity versus specificity for three models: combination (blue line), CK (green line), and HOTTIP (red line). The reference line (AUC = 0.5) indicates no discriminative ability. Sensitivity (true positive rate) is plotted on the *Y*-axis, while 1—specificity (false positive rate) is plotted on the *X*-axis. Both metrics are expressed as normalized values ranging from 0 to 1. The combination model demonstrates the highest sensitivity and specificity, followed by HOTTIP and CK.

To assess the diagnostic potential of training effectiveness using a combination of the investigated biomarkers, logistic regression analysis was conducted alongside calculation of the area under the ROC curve (AUC; Table 7). The highest AUC value (0.970) was achieved by the model combining HOTTIP expression and CK levels. Detailed results are presented in Table 8 and Figure 3.

The addition of cortisol to this model did not significantly enhance its predictive performance as the model including HOTTIP, CK, and cortisol demonstrated a comparable AUC. Models combining HOTTIP and cortisol (AUC = 0.947) as well as CK and cortisol (AUC = 0.932) also exhibited robust classification performance, confirming the diagnostic utility of classical biochemical markers, albeit with slightly lower predictive power compared to models incorporating HOTTIP. The models based on the combination of HOTTIP and SNHG4 exhibited a high AUC of 0.903, suggesting potential synergistic effects between these long non-coding RNAs.

## 3. Discussion

In this study, we showed for the first time that lncRNA HOTTIP demonstrated a notable increase in expression during the training period, as well as and in combination with CK, meaning it can be used as a potential marker of training efficiency. This marked upregulation suggests that HOTTIP is induced by chronic exercise stimuli, indicating its potentially crucial role in the long-term physiological adaptation to intensive physical training.

HOTTIP (HOXA transcript at the distal tip) is a well-established epigenetic regulator that activates the expression of genes within the HOXA cluster-key contributors to developmental processes, mesenchymal cell differentiation, tissue remodeling, and angiogenesis [31,32,33]. Previous reports [31,34] have shown that HOTTIP mediates the activation of *HOXA13* and *HOXA11* genes, which are essential for skeletal muscle regeneration and neovascularization in response to mechanical stress. In the context of elite athletes, elevated HOTTIP expression may reflect an adaptive response to repeated micro-injuries in muscle tissue and serve as a compensatory mechanism that promotes performance enhancement and tissue repair via structural and functional remodeling. Chronic physical exercise triggers complex biochemical and molecular cascades that rely on the activation of multiple signaling pathways. By regulating *HOXA* genes, HOTTIP may modulate several of these pathways, including PI3K/AKT and Wnt/β-catenin signaling, which are widely recognized as key regulators of cellular proliferation, differentiation, and angiogenesis [35,36]. Additional mechanistic evidence suggests a role for HOTTIP in the regulation of angiogenesis, a key process underlying long-term skeletal muscle adaptation. Studies by Liao et al., Zeng et al., and Wei et al. have demonstrated that HOTTIP promotes endothelial cell proliferation and migration through activation of the Wnt/β-catenin and HIF-1α/VEGF signaling pathways, thereby enhancing vascularization and tissue oxygenation in response to sustained mechanical loading. These activities align with the adaptive phenotype commonly observed in athletes undergoing intensive training [37,38,39].

The other analyzed lncRNAs—SNHG4, SNHG5, NEAT1, HIX003209, and PACERR—did not show significant changes in expression during the study period. Nevertheless, some displayed notable but inconsistent individual variations, potentially reflecting high inter-individual variability in molecular responses characteristic of athletic populations. Particularly noteworthy was the positive correlation observed between SNHG4 and HOTTIP expression levels. This co-variation may suggest co-regulation of these lncRNAs via shared transcriptional mechanisms or their involvement in related signaling pathways, warranting further investigation.

Particularly noteworthy are long non-coding RNAs belonging to the Small Nucleolar RNA Host Genes (SNHG) family, including SNHG4 and SNHG5. Numerous studies, primarily in the context of oncological diseases, have demonstrated that these transcripts can activate key cellular signaling pathways such as PI3K/AKT/mTOR and Wnt/β-catenin, which are among the most critical mechanisms regulating cell growth, differentiation, proliferation, and survival [22,23]. Although the majority of existing research on SNHG4 and SNHG5 focuses on their pathological roles (e.g., carcinogenesis, cell cycle regulation, or treatment resistance), the underlying mechanisms of their action are universal and also applicable to physiological processes of tissue remodeling and adaptation, particularly in skeletal muscle.

Muscle regeneration following microdamage induced by physical exertion, proliferation of satellite cells (muscle stem cells), and maintenance of metabolic homeostasis is a process regulated by the PI3K/AKT/mTOR and Wnt/β-catenin signaling pathways [19,20,21,40]. Within this framework, the transcripts SNHG4 and SNHG5 may play a regulatory role in the molecular mechanisms underpinning these physiological responses in the context of sports. Although changes in SNHG4 expression were not significant, a moderate increase might suggest possible biological relevance, especially in relation to regenerative and adaptive responses. SNHG4 is a lncRNA known to participate in the regulation of proliferative and apoptotic processes, and its interaction with HOTTIP could support the complex nature of transcriptomic-level adaptations. In our study, no significant differences in NEAT1 expression were observed between the two analyzed time points. NEAT1 is a key component of paraspeckles, nuclear substructures involved in regulating gene expression in response to cellular stress, including oxidative stress [24,25]. However, we did not observe a clear or consistent activation of NEAT1 following physical exercise. One possible explanation for this finding is the adaptive response to oxidative stress typical of trained individuals, which includes enhanced activity of antioxidant systems that may have attenuated the transcriptional response of NEAT1. Additionally, NEAT1 expression may be dynamic, potentially peaking within a narrower post-exercise window (e.g., within 2–6 h), which was not captured by the current study protocol and warrants further investigation with temporal resolution. It is also important to highlight that NEAT1 plays a critical role not only in stress response but also in the regulation of muscle cell proliferation. Skeletal muscle models have demonstrated that NEAT1 promotes myoblast proliferation, partly by suppressing the expression of the *Cdkn1a* gene, which encodes the cell cycle inhibitor p21CIP1 [17]. Based on findings by Wang et al., it was shown that NEAT1 significantly contributes to myogenesis by promoting myoblast proliferation and inhibiting their differentiation, suggesting that intense physical exercise might stimulate its expression [41]. Therefore, NEAT1 may potentially participate not only in stress responses but also in muscle remodeling and adaptive cell cycle regulation in individuals undergoing regular physical training. However, our results did not confirm a consistent pattern of NEAT1 expressions in response to exercise, which may be attributable to inter-individual variability, differences in training status, or compensatory mechanisms related to chronic adaptation to physiological stress.

The expression levels of the lncRNAs PACERR and HIX003209, both implicated in the regulation of inflammatory responses, were also analyzed. PACERR, which plays a crucial role in the transactivation of the *COX-2* gene promoter—a key pro-inflammatory enzyme—did not exhibit significant changes in expression levels following the training cycle [29,42]. Similarly, HIX003209 expression remained unchanged. This lncRNA is known to function as a competing endogenous RNA, exacerbating inflammation by “sponging” miR-6089 and activating the TLR4/NF-κB pathway in macrophages [43]. The absence of significant alterations in the expression of these transcripts may reflect adaptive regulation of the inflammatory response in trained individuals, wherein chronic physical activity attenuates inflammatory axis activation, an effect aligned with the concept of “immune quiescence” or “inflammaging” observed in athletes. Additionally, the timing of sample collection—covering the post-exercise recovery phase rather than the immediate acute response—may have contributed to the lack of detectable differences. For HIX003209, it is also possible that its expression is dependent on the type, intensity, and duration of the physiological stressor.

In our study, we observed that among the analyzed lncRNAs, only HIX003209 exhibited a significant negative correlation with the percentage change in creatine kinase levels following the training period. It is important to note that both absolute CK concentrations and percentage changes were assessed; however, while absolute CK levels did not show significant alterations, the percentage change proved to be a more sensitive indicator of muscle response to exercise. This approach accounts for individual baseline variability, which is crucial when evaluating the extent of muscle damage and the efficiency of recovery mechanisms. 

The observed negative correlation suggests that higher expression of HIX003209 may be associated with reduced muscle damage or enhanced protective and reparative processes. Consequently, HIX003209 might play a regulatory role in post-exercise muscle repair, positioning it as a promising candidate biomarker of muscle adaptation and a potential therapeutic target in muscle regeneration.

Creatine kinase is a widely used, cost-effective biomarker for assessing muscle damage and training load [44]. However, our results demonstrate that lncRNA HOTTIP not only correlates with CK levels but also provides additional molecular and epigenetic insights unattainable through CK measurement alone. HOTTIP exhibited the highest area under the ROC curve (AUC = 0.917) and a favorable Youden’s index (0.75), with sensitivity of 83% and specificity of 92%, confirming its strong discriminatory power in differentiating trained physiological states. Compared to classical markers such as CK (AUC = 0.894, Youden = 0.64, sensitivity 92%, and specificity 73%) and cortisol, HOTTIP demonstrated superior predictive performance. Logistic regression models revealed that combining HOTTIP with CK markedly improved diagnostic accuracy (AUC = 0.970), with a Youden’s index of 0.83, sensitivity of 83%, and specificity of 100%, making this combination a robust tool for monitoring training adaptation and detecting muscle overload.

The use of creatine kinase (CK) as a reliable biomarker in athletes is limited by its delayed response—peaking 24–72 h post-exercise—and short plasma half-life, which introduce variability depending on sampling time. Additionally, CK is a non-specific marker, responding to various forms of muscle damage, which restricts its ability to accurately reflect specific adaptive physiological processes [45,46,47]. In contrast, HOTTIP showed greater specificity and sensitivity, suggesting its utility as a biomarker for long-term physiological adaptation.

Although cortisol is frequently used to assess physiological stress responses, its pronounced circadian variation and susceptibility to external factors such as sleep quality, nutritional status, and psychological stress limit its effectiveness for tracking training progress. Moreover, chronic adaptation of the hypothalamic–pituitary–adrenal (HPA) axis to regular exercise may reduce cortisol sensitivity, complicating data interpretation in training evaluation.

Although CK still remains a valuable and economical marker of muscle damage, integrating HOTTIP into training monitoring protocols significantly enhances the precision and sensitivity of physiological adaptation assessment, enabling earlier detection of molecular adaptation signals and overload. The combined use of these biomarkers facilitates a comprehensive, multidimensional evaluation of an athlete’s physiological status, supporting optimized and individualized training regimens.

SNHG4 stood out with perfect specificity (100%) and moderate sensitivity (75%) at an AUC of 0.785, indicating its potential as a complementary marker, especially when combined with HOTTIP, where their combined AUC reached 0.903, suggesting a synergistic effect. This indicates an important role for HOTTIPA as a potential biomarker of training adaptation.

Although VO_2_ max is a parameter used to assess aerobic capacity, it demonstrated perfect sensitivity (100%) in the examined model; however, its specificity was low (33%), limiting its practical diagnostic utility. While VO_2_ max is widely regarded as a classical indicator of endurance performance, it does not always precisely reflect the current state of training adaptation. This is because improvements in VO_2_ max primarily result from long-term cardiovascular and respiratory adaptations rather than immediate effects of recent training sessions. Such changes occur gradually and often do not correlate with short-term fluctuations in performance or recovery status [48]. The lack of significant changes in VO_2_ max observed in our study after 10 weeks of training aligns with these limitations. This duration may be insufficient to detect meaningful modifications, especially if training intensity or volume is inadequate. Therefore, there is a clear necessity to incorporate more sensitive and specific molecular biomarkers that enable more accurate and timely monitoring of physiological changes associated with exercise adaptation.

It is important to emphasize that this study has a pilot character due to its limited sample size. Nevertheless, the consistent and significant changes in HOTTIP expression highlight important biological trends that justify further, more extensive research. The pilot nature of this study allows for preliminary assessment of the biomarker’s potential and optimization of study protocols before implementation in larger cohorts. In this study, lncRNA expression was evaluated at two strategically selected time points: immediately following the summer break (baseline) and upon completion of a 10-week training regimen (endpoint). This temporal framework was intentionally designed to capture sustained, cumulative molecular adaptations associated with the entire training cycle, rather than transient transcriptional responses elicited by individual exercise sessions. Recent studies showed that single and acute sessions of exercise are known to elicit rapid yet transient alterations in gene expression. These molecular changes predominantly reflect the immediate physiological response to exercise-induced oxidative stress, perturbations in cellular homeostasis, and the activation of signaling cascades associated with tissue repair and adaptation. While biologically significant, such ephemeral transcriptional shifts may not necessarily contribute to long-term adaptive outcomes and can complicate the interpretation of molecular data in the context of chronic training-induced adaptations [1,49,50]. Future research should focus on larger participant groups, longitudinal expression analyses at multiple time points (immediately post-exercise and during recovery), and integration of transcriptomic data with proteomic, cytokine, hormonal, and metabolomic profiles. Our previous study [34] did not reveal significant differences in IL-6 levels between the baseline and endpoint measurements, which allows us to exclude inflammation as a potential factor influencing lncRNAs expression. However, it is important to acknowledge that other inflammatory markers should also be considered to comprehensively rule out the potential impact of subclinical or systemic inflammatory responses on gene expression. Moreover, controlling for variables such as exercise modality, training volume, and physiological factors (e.g., menstrual cycle phase in females) is essential to better understand the influences on transcriptomic responses and improve the specificity and applicability of lncRNA biomarkers in training monitoring. Finally, the results should be validated in a control group comprising individuals who do not engage in professional training in order to determine whether the observed effects are specific to trained individuals or generalizable to the broader population. Therefore, further investigations incorporating diverse exercise models and temporal expression analyses are warranted to definitively elucidate the roles of these lncRNAs in exercise-induced adaptation.

## 4. Materials and Methods

### 4.1. Subject and Study Design

This study constitutes an extension of previous research aimed at identifying potential biomarkers of training adaptation and physiological changes in elite female volleyball athletes. The study was approved by the Bioethics Committee of the University of Rzeszów (Protocol No. 3/11/2017).

The study was conducted on elite professional female volleyball players who are national team representatives and compete at the highest levels of domestic and international leagues. The entire team was included during a real-life preparatory period following the summer break, ensuring the consistency and representativeness of the study population. The participants had an average training experience of 13 years and 8 months (±6 years and 5 months).

Baseline anthropometric and demographic characteristics were collected and have been described in detail in earlier studies. Physiological and biochemical data used in the present study were obtained from a previous publication [5,30]. These included a mean age of 27 ± 5.4 years (mean ± SD), a mean height of 184.61 ± 9.37 cm, and a mean body weight of 76.27 ± 12.76 kg. All participants were injury-free and reported no use of pharmacological agents during the study period. Written informed consent was obtained from all subjects prior to enrollment. Due to strict inclusion criteria and limited availability of this highly specialized group—resulting from a demanding competition schedule and national team commitments—the sample size was limited, which is typical for studies involving world-class athletes [51,52,53,54,55]. The intervention consisted of a 10-week training program comprising three distinct phases: a 2-week introductory phase, a 6-week preparatory phase, and a 2-week specialized training phase.

The study included two key time points: a baseline measurement conducted immediately after the summer break, serving as a natural control condition, and an endpoint measurement at the conclusion of the preparatory period. This design allowed us to exclude the influence of other physiological states and assess biomarker changes during the intensive preseason training phase.

During the preparatory period, athletes completed 131 training sessions across 10 weekly microcycles. The program included 2 introductory weeks, 6 weeks of general preparation, and 2 weeks of specialized training. Training load distribution evolved over time: weeks 1–3 focused on aerobic (80%) and strength-resistance (20%) work; weeks 4–6 balanced aerobic (60%) and resistance (40%) efforts; and weeks 7–10 emphasized power training (80%) with reduced aerobic input (20%). A total of 13,100 min (218.3 h) were devoted to training, with the highest load in microcycle 4 (1800 min) and the lowest in microcycle 8 (780 min). Additionally, 1320 min were allocated to recovery methods. Details were previously described. The methodology for the training intervention, as well as the biochemical and physiological assessments, have been previously described in detail [5,30].

Throughout the studied period, regular assessments were conducted to monitor physiological and biochemical responses to training. These included measurements of maximal oxygen uptake (VO_2_ max) and blood concentrations of creatine kinase and cortisol, as well as body composition parameters. VO_2_ max was estimated using a standardized, field-based incremental running test designed to progressively evaluate aerobic performance under practical conditions. Venous blood samples were collected from the median cubital vein into 2 mL EDTA-coated tubes at two time points: in the morning at baseline and at the conclusion of the 10-week training cycle.

### 4.2. Expression Analysis

For gene expression analysis, one milliliter of whole blood was used. Biological samples were consistently collected at the same time of day to control for circadian influences. Erythrocytes were lysed using 1× RBC Lyse Buffer (EURx, Gdańsk, Poland) at a 4:1 ratio and placed on ice. The mixture was then centrifuged at 400× g for 10 min. The resulting pellet was washed with 5 mL of 1× phosphate-buffered saline (PBS; EURx, Gdańsk, Poland), centrifuged again, and subsequently rinsed with PBS before a final centrifugation step. The supernatant was discarded. Next, 1 mL of RNA Extracol (EURx, Gdańsk, Poland) was added, and the samples were stored at −80 °C until RNA isolation. Total RNA was extracted using the chloroform–isopropanol method (Chempur, Piekary Śląskie, Poland), as previously described by Foroni et al. [56].

The extracted RNA was resuspended in 15 µL of RNase-free water in few aliquots and stored at −80 °C until further analysis. Before reverse transcription, total RNA concentration was evaluated with the Qubit 4 instrument using the Qubit RNA Quantitation High Sensitivity Assay Kit (Thermo Fisher Scientific, Waltham, MA, USA). The RT reaction was performed using 300 ng of total RNA in a 20 μL reaction mixture with the smART RT-PCR Kit (EURx, Gdańsk Poland), following the manufacturer’s instructions and using a two-step cDNA synthesis protocol. In the first step, 10.5 μL of appropriately diluted RNA was mixed with 0.5 μL of Oligo (dT) primer (50 μM), 0.5 μL of random hexamer primer (200 ng/μL), and 1 μL of dNTP mix (10 mM). In the second step, 4 μL of 5× cDNA buffer, 2 μL of DTT (0.1 M), 0.5 μL of RNase inhibitor (50 U/μL), and 1 μL of smART enzyme (200 U/μL) were added. The resulting complementary DNA (cDNA) was stored at −20 °C until further analysis. Prior to gene expression quantification, cDNA was diluted twofold to a final concentration of 150 ng/μL for all analyzed transcripts. The levels of lncRNAs SNHG4, SNHG5, NEAT1, HIX003209, HOTTIP, and PACERR were quantified from cDNA using specific primers by real-time PCR with the SG qPCR Master Mix (EURx, Gdańsk, Poland) in accordance with the manufacturer’s protocol. The primer sequences used in this study were adapted from the publication by Cieśla M. et al. [29] with the exception of SNHG4 and SNHG5. SNHG4 primers had the following sequences (5′→3′): forward: GCACTAAGGTGACTGCAAGAA, reverse: ACTGAGATTGTGGCTGAGTTTG. SNHG5 primers had the following sequences (5′→3′): forward: CTGAGTGTGGACGAGTAGCC, reverse: TATCAATGGCAGACAGCGA and were designed using the Primer-BLAST online tool [18]. All primers were used at a final concentration of 450 nM. Amplification was carried out using the QuantStudio Real-Time PCR System (Applied Biosystems, Waltham, MA, USA). The PCR conditions were as follows: holding stage at 50 °C, initial denaturation at 95 °C, followed by 45 amplification cycles consisting of denaturation at 94 °C for 15 s, annealing at 60 °C for 30 s, and extension at 72 °C for 30 s. Melting curve analysis was performed following each amplification. All samples were evaluated in triplicate. The specificity of the PCR products was assessed by electrophoresis on a 1.5% agarose gel. A calibrator sample, prepared by pooling cDNA from randomly selected samples, was used as a reference. The cDNA was aliquoted and frozen to prevent repeated freeze–thaw cycles. Subsequently, aliquots of the calibrator cDNA were added to each reaction plate to normalize and eliminate inter-plate variability. Quantitative determination of lncRNA expression was performed using the comparative Ct method (ΔΔCt) in QuantStudio Design and Analysis Software version 1.5.2 (Applied Biosystems, Thermo Fisher Scientific, Waltham, MA, USA). Gene expression results were calculated as relative quantitative measurements (RQ) and presented as fold changes. The glyceraldehyde-3-phosphate dehydrogenase (*GAPDH*) gene was used as a reference for normalization of gene expression levels.

### 4.3. Statistics

The distribution of quantitative variables was assessed using the Shapiro–Wilk test. Data with a normal distribution are presented as means ± standard deviation (SD), while non-normally distributed variables are expressed as medians with interquartile ranges (IQR; 25th–75th percentile). Differences between two time points were analyzed using repeated measures analysis of variance (ANOVA). Multiple testing correction was performed using the Benjamini–Hochberg procedure to control the false discovery rate (FDR). Correlations between continuous variables were assessed using Spearman’s rank correlation coefficient. Receiver operating characteristic (ROC) curve analysis was conducted to determine the Youden index (YI), sensitivity, and specificity of selected variables. The diagnostic utility of potential biomarkers was further assessed using logistic regression, with model performance expressed as the area under the curve (AUC). A *p*-value of less than 0.05 was considered statistically significant. All statistical analyses were performed using STATISTICA software, version 13.3 (TIBCO Software Inc., Palo Alto, CA, USA).

## 5. Conclusions

In conclusion, our findings clearly demonstrate that lncRNA HOTTIP plays a crucial role in muscle adaptation to chronic physical training, exhibiting a significant upregulation. HOTTIP emerges as a unique and highly precise biomarker of training adaptation, providing additional molecular and epigenetic insights beyond those offered by traditional markers such as creatine kinase. Its high sensitivity and specificity indicate that incorporating HOTTIP measurement could substantially improve the monitoring and optimization of training processes, particularly in elite athlete populations. However, further studies involving larger, more representative cohorts are necessary to validate its practical application and integration into standard training assessment protocols.

Based on these results, we propose a preliminary diagnostic algorithm for evaluating physiological adaptation to chronic exercise, using lncRNA HOTTIP expression as the primary biomarker. This algorithm involves the simultaneous assessment of HOTTIP expression levels and serum creatine kinase (CK) concentrations. Elevated HOTTIP expression, especially when accompanied by a moderate increase in CK, suggests effective adaptation characterized by favorable regenerative processes and enhanced physiological performance. Conversely, low HOTTIP expression combined with high CK levels may indicate muscle overload and insufficient recovery, while high HOTTIP expression with minimal CK elevation suggests superior adaptive capacity and effective muscle protection mechanisms. These results may serve as a starting point for further extended research encompassing diverse athlete groups and allowing for validation and generalization of the observed relationships.

## Figures and Tables

**Table 1:** Changes in the expression levels of selected lncRNAs and biochemical parameters between baseline and endpoint.

Parameter	Baseline	Endpoint	*p*-Value	Adjusted
SNHG4	0.53 [0.27–0.74]	1.45 [0.75–4.62]	0.149	0.298
SNHG5	0.96 ± 0.41	0.83 ± 0.55	0.214	0.285
NEAT1	1.12 [0.70–1.74]	1.47 [0.76–1.99]	0.773	0.773
HIX003209	1.44 [0.94–2.06]	0.97 [0.81–1.25]	0.149	0.238
PACERR	1.3 [0.7–2.25]	1.12 [0.71–5.29]	0.773	0.773
HOTTIP	0.3 [0.62–0.63]	2.02 [1.3–6.57]	**0.009**	**0.024**

Data are presented as fold change and expressed as mean ± SD or median [lowerupper quartile]. *p*-values were estimated using repeated measures ANOVA. Significant differences are indicated in bold.

**Table 2:** Baseline correlation between relative lncRNA expression and body composition and training parameters.

Parameter	Weight	BMI	BMR	FAT%	Fat Mass	VO_2_ Max.	CK	CK%	CORT
SNHG4	0.27	0.31	0.06	**0.69**	0.57	−0.33	0.09	−0.12	0.24
SNHG5	0.48	0.27	0.52	0.06	0.27	−0.54	0	0	0.23
NEAT1	−0.37	−0.44	−0.32	−0.24	−0.28	0.11	−0.21	−0.13	0.18
HIX003209	0.22	0.25	−0.03	0.46	0.41	−0.18	0	−0.31	**0.62**
HOTTIP	0.34	0.34	0.18	0.53	0.49	−0.31	0.1	−0.04	−0.05
PACERR	0.11	0.33	0.03	0.16	0.18	−0.15	0.08	0.18	0.41

Abbreviations: BMI—Body Mass Index, BMR—Basal Metabolic Rate, FAT%—percentage of body fat, VO_2_ max—maximal oxygen uptake, CK—creatine kinase (a biomarker of muscle damage), CK%—percentage change in creatine kinase, and CORT—cortisol (a glucocorticoid stress hormone). The terms SNHG4, SNHG5, NEAT1, HIX003209, HOTTIP, and PACERR denote the specific long non-coding RNAs (lncRNAs) evaluated in this study. Significant correlations are highlighted in bold.

**Table 3:** Endpoint correlation between relative lncRNA expressions and body composition and training parameters.

Parameter	Weight	BMI	BMR	FAT%	Fat Mass	VO_2_ Max.	CK	CK%	CORT
SNHG4	0.14	−0.29	0.1	0.03	0.08	0.07	0.03	0.08	0.03
SNHG5	0.43	0.21	0.49	0.2	0.31	−0.32	0.2	0.31	0.2
NEAT1	0.34	−0.01	0.27	0.42	0.49	−0.38	0.42	0.49	0.42
HIX003209	−0.29	−0.02	−0.34	−0.07	−0.08	0.12	−0.07	**−0.8**	−0.07
HOTTIP	−0.02	−0.39	−0.15	0.17	0.06	0.17	0.17	0.06	0.17
PACERR	−0.07	−0.2	−0.27	0.34	0.28	0.01	0.34	0.28	0.34

Abbreviations: BMI—Body Mass Index, BMR—Basal Metabolic Rate, FAT%—percentage of body fat, VO_2_ max—maximal oxygen uptake, CK—creatine kinase (a biomarker of muscle damage), CK%—percentage change in creatine kinase, and COR—cortisol (a glucocorticoid stress hormone). The terms SNHG4, SNHG5, NEAT1, HIX003209, HOTTIP, and PACERR denote the specific long non-coding RNAs (lncRNAs) evaluated in this study. Significant correlations are highlighted in bold.

**Table 4:** Correlation between relative lncRNA expression levels at baseline.

	SNHG4	SNHG5	NEAT1	HIX003209	HOTTIP	PACERR
SNHG4	1					
SNHG5	−0.18	1				
NEAT1	−0.12	0.02	1			
HIX003209	**0.71**	0.08	−0.38	1		
HOTTIP	**0.89**	−0.24	−0.37	0.55	1	
PACERR	0.37	−0.16	−0.4	**0.59**	0.38	1

Significant correlations are highlighted in bold.

**Table 5:** Correlation between relative lncRNA expression levels at endpoint.

	SNHG4	SNHG5	NEAT1	HIX003209	HOTTIP	PACERR
SNHG4	1					
SNHG5	0.41	1				
NEAT1	**0.83**	0.49	1			
HIX003209	−0.12	0.33	−0.14	1		
HOTTIP	**0.7**	−0.16	**0.58**	−0.43	1	
PACERR	0.48	−0.11	0.52	0.26	**0.59**	1

Significant correlations are highlighted in bold.

**Table 6:** Evaluation of the diagnostic potential and usefulness of the studied parameters in assessing training effectiveness.

	AUC	*p*-Value	Youden’s Index	Sensitivity [%]	Specificity [%]
CK	0.894	**<0.001**	0.64	92	73
CORT	0.856	**<0.001**	0.73	100	73
VO_2_ max	0.678	0.116	0.33	100	33
SNHG4	0.785	**0.011**	0.75	75	100
SNHG5	0.556	0.649	0.25	25	100
NEAT1	0.549	0.691	0.25	42	83
HIX003209	0.712	0.055	0.5	100	25
HOTTIP	0.917	**<0.001**	0.75	83	92
PACERR	0.59	0.429	0.33	33	100

Abbreviations: AUC—area under the curve; CK—creatine kinase, CORT—cortisol; for others, please refer to previous tables. Significant *p*-values are in bold.

**Table 7:** Assessment of the diagnostic value of the targets studied using a logistic regression model.

Variable	AUC
CK + CORT	0.932
HOTTIP	0.917
HOTTIP + CK	**0.970**
HOTTIP + CORT	0.947
HOTTIP + CK+CORT	**0.970**
HOTTIP + SNHG4	0.903

Abbreviations: AUC—area under the curve; CK—creatine kinase, CORT—cortisol. The strongest AUCs are indicated in bold.

**Table 8:** Comparison of diagnostic accuracy for CK, HOTTIP, and combined model.

	AUC	*p*-Value	Youden’s Index	Sensitivity [%]	Specificity [%]	Cut-Off Value
CK	0.894	<0.001	0.64	92	73	126
HOTTIP	0.917	**<0.001**	0.75	83	92	1.23
Combination	0.97	**<0.001**	0.83	83	100	2.086

Abbreviations: AUC—area under the curve; CK—creatine kinase. Combination refers to jointly using CK and HOTTIP in a diagnostic model. Statistically significant *p*-values and highest specificity values are highlighted in bold.

## Data Availability

The data generated in this publication are available from the corresponding author upon reasonable request.

## References

[1] Egan B., O’Connor P.L., Zierath J.R., O’Gorman D.J. (2013). Time Course Analysis Reveals Gene-Specific Transcript and Protein Kinetics of Adaptation to Short-Term Aerobic Exercise Training in Human Skeletal Muscle. PLoS ONE.

[2] Crewther B.T., Cook C., Cardinale M., Weatherby R.P., Lowe T. (2011). Two Emerging Concepts for Elite Athletes: The Short-Term Effects of Testosterone and Cortisol on the Neuromuscular System and the Dose-Response Training Role of These Endogenous Hormones. Sports Med..

[3] Egan B., Zierath J.R. (2013). Exercise Metabolism and the Molecular Regulation of Skeletal Muscle Adaptation. Cell Metab..

[4] Polakovičová M., Musil P., Laczo E., Hamar D., Kyselovič J. (2016). Circulating MicroRNAs as Potential Biomarkers of Exercise Response. Int. J. Mol. Sci..

[5] Podgórski R., Cieśla M., Podgórska D., Bajorek W., Płonka A., Czarny W., Trybulski R., Król P. (2022). Plasma microRNA-320a as a Potential Biomarker of Physiological Changes during Training in Professional Volleyball Players. J. Clin. Med..

[6] Coffey V.G., Hawley J.A. (2007). The Molecular Bases of Training Adaptation. Sports Med..

[7] Soci U.P.R., Melo S.F.S., Gomes J.L.P., Silveira A.C., Nóbrega C., de Oliveira E.M. (2017). Exercise Training and Epigenetic Regulation: Multilevel Modification and Regulation of Gene Expression. Adv. Exp. Med. Biol..

[8] Widmann M., Nieß A.M., Munz B. (2019). Physical Exercise and Epigenetic Modifications in Skeletal Muscle. Sports Med..

[9] Voisin S., Eynon N., Yan X., Bishop D.J. (2015). Exercise Training and DNA Methylation in Humans. Acta Physiol..

[10] De Sanctis P., Filardo G., Abruzzo P.M., Astolfi A., Bolotta A., Indio V., Di Martino A., Hofer C., Kern H., Löfler S. (2021). Non-Coding RNAs in the Transcriptional Network That Differentiates Skeletal Muscles of Sedentary from Long-Term Endurance- and Resistance-Trained Elderly. Int. J. Mol. Sci..

[11] (2004). The ENCODE (ENCyclopedia Of DNA Elements) Project. Science.

[12] Walter N.G. (2024). Are Non-Protein Coding RNAs Junk or Treasure?. BioEssays.

[13] Good D.J. (2023). Non-Coding RNAs in Human Health and Diseases. Genes.

[14] Brown C.J., Ballabio A., Rupert J.L., Lafreniere R.G., Grompe M., Tonlorenzi R., Willard H.F. (1991). A Gene from the Region of the Human X Inactivation Centre Is Expressed Exclusively from the Inactive X Chromosome. Nature.

[15] Iyer M.K., Niknafs Y.S., Malik R., Singhal U., Sahu A., Hosono Y., Barrette T.R., Prensner J.R., Evans J.R., Zhao S. (2015). The Landscape of Long Noncoding RNAs in the Human Transcriptome. Nat. Genet..

[16] Bonilauri B., Dallagiovanna B. (2020). Long Non-Coding RNAs Are Differentially Expressed After Different Exercise Training Programs. Front. Physiol..

[17] Archacka K., Ciemerych M.A., Florkowska A., Romanczuk K. (2021). Non-Coding RNAs as Regulators of Myogenesis and Postexercise Muscle Regeneration. Int. J. Mol. Sci..

[18] Ye J., Coulouris G., Zaretskaya I., Cutcutache I., Rozen S., Madden T.L. (2012). Primer-BLAST: A Tool to Design Target-Specific Primers for Polymerase Chain Reaction. BMC Bioinform..

[19] Otto A., Schmidt C., Luke G., Allen S., Valasek P., Muntoni F., Lawrence-Watt D., Patel K. (2008). Canonical Wnt Signalling Induces Satellite-Cell Proliferation during Adult Skeletal Muscle Regeneration. J. Cell Sci..

[20] Glass D.J., Rommel C., Vanhaesebroeck B., Vogt P.K. (2011). PI3 Kinase Regulation of Skeletal Muscle Hypertrophy and Atrophy. Phosphoinositide 3-Kinase in Health and Disease: Volume 1.

[21] Han X.H., Jin Y.-R., Seto M., Yoon J.K. (2011). A WNT/β-Catenin Signaling Activator, R-Spondin, Plays Positive Regulatory Roles during Skeletal Myogenesis*. J. Biol. Chem..

[22] Zimta A.-A., Tigu A.B., Braicu C., Stefan C., Ionescu C., Berindan-Neagoe I. (2020). An Emerging Class of Long Non-Coding RNA With Oncogenic Role Arises From the snoRNA Host Genes. Front. Oncol..

[23] Hu X., Zhang Q., Xing W., Wang W. (2022). Role of microRNA/lncRNA Intertwined With the Wnt/β-Catenin Axis in Regulating the Pathogenesis of Triple-Negative Breast Cancer. Front. Pharmacol..

[24] Hirose T., Virnicchi G., Tanigawa A., Naganuma T., Li R., Kimura H., Yokoi T., Nakagawa S., Bénard M., Fox A.H. (2014). NEAT1 Long Noncoding RNA Regulates Transcription via Protein Sequestration within Subnuclear Bodies. Mol. Biol. Cell.

[25] Doerner S.K., Reis E.S., Leung E.S., Ko J.S., Heaney J.D., Berger N.A., Lambris J.D., Nadeau J.H. (2016). High-Fat Diet-Induced Complement Activation Mediates Intestinal Inflammation and Neoplasia, Independent of Obesity. Mol. Cancer Res..

[26] Peake J.M., Neubauer O., Della Gatta P.A., Nosaka K. (2017). Muscle Damage and Inflammation during Recovery from Exercise. J. Appl. Physiol..

[27] Liu Y., Wang X., Zhu Y., Cao Y., Wang L., Li F., Zhang Y., Li Y., Zhang Z., Luo J. (2022). The CTCF/LncRNA-PACERR Complex Recruits E1A Binding Protein P300 to Induce Pro-tumour Macrophages in Pancreatic Ductal Adenocarcinoma via Directly Regulating PTGS2 Expression. Clin. Transl. Med..

[28] Docherty S., Harley R., McAuley J.J., Crowe L.A.N., Pedret C., Kirwan P.D., Siebert S., Millar N.L. (2022). The Effect of Exercise on Cytokines: Implications for Musculoskeletal Health: A Narrative Review. BMC Sports Sci. Med. Rehabil..

[29] Cieśla M., Darmochwał-Kolarz D., Pałka A., Łagowska K., Tabarkiewicz J., Kolarz B. (2024). Expression of lncRNAs NEAT1, PACERR, and GAS5 Can Be Associated with Disease Activity in Rheumatoid Arthritis Patients. Pol. Arch. Intern. Med..

[30] Podgórska D., Cieśla M., Płonka A., Bajorek W., Czarny W., Król P., Podgórski R. (2024). Changes in Circulating MicroRNA Levels as Potential Indicators of Training Adaptation in Professional Volleyball Players. Int. J. Mol. Sci..

[31] Ye Y., Li Y., Wei Y., Xu Y., Wang R., Fu Z., Zheng S., Zhou Q., Zhou Y., Chen R. (2018). Anticancer Effect of HOTTIP Regulates Human Pancreatic Cancer via the Metabotropic Glutamate Receptor 1 Pathway. Oncol. Lett..

[32] Xiao Z.S., Long H., Zhao L., Li H.X., Zhang X.N. (2020). LncRNA HOTTIP Promotes Proliferation and Inhibits Apoptosis of Gastric Carcinoma Cells via Adsorbing miR-615-3p. Eur. Rev..

[33] Han L., Yan Y., Zhao L., Liu Y., Lv X., Zhang L., Zhao Y., Zhao H., He M., Wei M. (2020). LncRNA HOTTIP Facilitates the Stemness of Breast Cancer via Regulation of miR-148a-3p/WNT1 Pathway. J. Cell. Mol. Med..

[34] Sun Y., Zhou Y., Bai Y., Wang Q., Bao J., Luo Y., Guo Y., Guo L. (2017). A Long Non-Coding RNA HOTTIP Expression Is Associated with Disease Progression and Predicts Outcome in Small Cell Lung Cancer Patients. Mol. Cancer.

[35] Kalder M., Kyvernitakis I., Albert U.S., Baier-Ebert M., Hadji P. (2015). Effects of Zoledronic Acid versus Placebo on Bone Mineral Density and Bone Texture Analysis Assessed by the Trabecular Bone Score in Premenopausal Women with Breast Cancer Treatment-Induced Bone Loss: Results of the ProBONE II Substudy. Osteoporos. Int..

[36] Gokal K., Wallis D., Ahmed S., Boiangiu I., Kancherla K., Munir F. (2016). Effects of a Self-Managed Home-Based Walking Intervention on Psychosocial Health Outcomes for Breast Cancer Patients Receiving Chemotherapy: A Randomised Controlled Trial. Support. Care Cancer.

[37] Liao B., Chen R., Lin F., Mai A., Chen J., Li H., Xu Z., Dong S. (2018). Long Noncoding RNA HOTTIP Promotes Endothelial Cell Proliferation and Migration via Activation of the Wnt/β-Catenin Pathway. J. Cell. Biochem..

[38] Zeng X., Dong Q., Liu Q., Tan W.-J., Liu X.-D. (2022). LncRNA HOTTIP Facilitates Osteogenic Differentiation in Bone Marrow Mesenchymal Stem Cells and Induces Angiogenesis via Interacting with TAF15 to Stabilize DLX2. Exp. Cell Res..

[39] Wei H., Xu Z., Chen L., Wei Q., Huang Z., Liu G., Li W., Wang J., Tang Q., Pu J. (2022). Long Non-Coding RNA PAARH Promotes Hepatocellular Carcinoma Progression and Angiogenesis via Upregulating HOTTIP and Activating HIF-1α/VEGF Signaling. Cell Death Dis..

[40] Chu Q., Gu X., Zheng Q., Guo Z., Shan D., Wang J., Zhu H. (2021). Long Noncoding RNA SNHG4: A Novel Target in Human Diseases. Cancer Cell Int..

[41] Wang S., Zuo H., Jin J., Lv W., Xu Z., Fan Y., Zhang J., Zuo B. (2019). Long Noncoding RNA Neat1 Modulates Myogenesis by Recruiting Ezh2. Cell Death Dis..

[42] Desind S.Z., Iacona J.R., Yu C.Y., Mitrofanova A., Lutz C.S. (2022). PACER lncRNA Regulates COX-2 Expression in Lung Cancer Cells. Oncotarget.

[43] Yan S., Wang P., Wang J., Yang J., Lu H., Jin C., Cheng M., Xu D. (2019). Long Non-Coding RNA HIX003209 Promotes Inflammation by Sponging miR-6089 via TLR4/NF-κB Signaling Pathway in Rheumatoid Arthritis. Front. Immunol..

[44] Hacker S., Keck J., Reichel T., Eder K., Ringseis R., Krüger K., Krüger B. (2023). Biomarkers in Endurance Exercise: Individualized Regulation and Predictive Value. Transl. Sports Med..

[45] Brancaccio P., Lippi G., Maffulli N. (2010). Biochemical Markers of Muscular Damage. Clin. Chem. Lab. Med..

[46] Baird M.F., Graham S.M., Baker J.S., Bickerstaff G.F. (2012). Creatine-Kinase- and Exercise-Related Muscle Damage Implications for Muscle Performance and Recovery. J. Nutr. Metab..

[47] Mougios V. (2007). Reference Intervals for Serum Creatine Kinase in Athletes. Br. J. Sports Med..

[48] De Revere J.L., Clausen R.D., Astorino T.A. (2021). Changes in VO2max and Cardiac Output in Response to Short-Term High-Intensity Interval Training in Caucasian and Hispanic Young Women: A Pilot Study. PLoS ONE.

[49] Perry C.G.R., Lally J., Holloway G.P., Heigenhauser G.J.F., Bonen A., Spriet L.L. (2010). Repeated Transient mRNA Bursts Precede Increases in Transcriptional and Mitochondrial Proteins during Training in Human Skeletal Muscle. J. Physiol..

[50] Murton A.J., Billeter R., Stephens F.B., Des Etages S.G., Graber F., Hill R.J., Marimuthu K., Greenhaff P.L. (2014). Transient Transcriptional Events in Human Skeletal Muscle at the Outset of Concentric Resistance Exercise Training. J. Appl. Physiol. Bethesda Md 1985.

[51] Martínez A.C., Seco Calvo J., Tur Marí J.A., Abecia Inchaurregui L.C., Orella E.E., Biescas A.P. (2010). Testosterone and Cortisol Changes in Professional Basketball Players through a Season Competition. J. Strength Cond. Res..

[52] Lima R.F., Palao J.M., Clemente F.M. (2019). Jump Performance During Official Matches in Elite Volleyball Players: A Pilot Study. J. Hum. Kinet..

[53] McCall A., Wolfberg A., Ivarsson A., Dupont G., Larocque A., Bilsborough J. (2023). A Qualitative Study of 11 World-Class Team-Sport Athletes’ Experiences Answering Subjective Questionnaires: A Key Ingredient for ‘Visible’ Health and Performance Monitoring?. Sports Med..

[54] Miloski B., de Freitas V.H., Nakamura F.Y., de A Nogueira F.C., Bara-Filho M.G. (2016). Seasonal Training Load Distribution of Professional Futsal Players: Effects on Physical Fitness, Muscle Damage and Hormonal Status. J. Strength Cond. Res..

[55] Horta T.A.G., Bara Filho M.G., Coimbra D.R., Miranda R., Werneck F.Z. (2019). Training Load, Physical Performance, Biochemical Markers, and Psychological Stress During a Short Preparatory Period in Brazilian Elite Male Volleyball Players. J. Strength Cond. Res..

[56] Foroni L., Wilson G., Gerrard G., Mason J., Grimwade D., White H.E., de Castro D.G., Austin S., Awan A., Burt E. (2011). Guidelines for the Measurement of BCR-ABL1 Transcripts in Chronic Myeloid Leukaemia. Br. J. Haematol..

